# Rapid screening of ancient DNA extracts through uracil detection

**DOI:** 10.1016/j.isci.2026.117820

**Published:** 2026-10-15

**Authors:** Nihan Dilşad Dağtaş, Viktória Perey-Simon, Fabrizio Mafessoni, Dheeraj Chaudhary, Azhar Jabareen, Aviva Lindell, Valentina Vanghi, Krzysztof Cyrek, Andrew W. Kandel, Maciej T. Krajcarz, Magdalena Krajcarz, György Lengyel, Manuel Ángel Rojo-Guerra, Shmuel Safra, Policarpo Sánchez-Yustos, Ron Shimelmitz, Magdalena Sudoł-Procyk, Jarosław Wilczyński, Eszter Holub, Angéla Békési, Beáta G. Vértessy, Viviane Slon

**Affiliations:** 1 Department of Anatomy and Biological Anthropology and Department of Human Genetics and Computational Medicine, The Gray Faculty of Health & Medical Sciences, Tel Aviv University, Tel Aviv 6997801, Israel; 2 The Dan David Center for Human Evolution and Biohistory Research, Tel Aviv University, Tel Aviv 6997801, Israel; 3 Department of Applied Biotechnology and Food Science, Faculty of Chemical Technology and Biotechnology, Budapest University of Technology and Economics, 1111 Budapest, Hungary; 4 Genome Metabolism Research Group, Institute of Molecular Life Sciences, HUN-REN Research Centre for Natural Sciences, 1117 Budapest, Hungary; 5 Dipartimento di Scienze della Vita, Università di Trieste, 34127 Trieste, Italy; 6 Institute of Archaeology, Nicolaus Copernicus University, 87-100 Toruń, Poland; 7 The Role of Culture in Early Expansions of Humans, Heidelberg Academy of Sciences and Humanities at the University of Tübingen, Hölderlinstr. 12, 72074 Tübingen, Germany; 8 Institute of Geological Sciences, Polish Academy of Sciences, 00-901 Warszawa, Poland; 9 Hungarian National Museum Public Collection Centre, Múzeum krt 14-16, 1088 Budapest, Hungary; 10 Department of Archaeology, University of Miskolc, 3515 Miskolc, Hungary; 11 Área de Prehistoria, Departamento de Prehistoria, Arqueología, Antropología Social y Ciencias y Técnicas Historiográficas, University of Valladolid, 47011 Valladolid, Spain; 12 Faculty of Exact Sciences, School of Mathematical Sciences, Tel Aviv University, Tel Aviv 6997801, Israel; 13 Zinman Institute of Archaeology, School of Archaeology and Maritime Cultures, University of Haifa, Mount Carmel, Haifa 3498838, Israel; 14 Institute of Systematics and Evolution of Animals, Polish Academy of Sciences, Sławkowska 17, 31-016 Kraków, Poland

**Keywords:** ancient DNA, cytosine deamination, uracil-DNA, sedimentary ancient DNA, dot-blot assay, uracil detection

## Abstract

Paleogenetic studies provide invaluable insights into past populations. However, the success of ancient DNA retrieval remains difficult to predict. Ancient DNA workflows are time consuming and costly, motivating methods to assess preservation at an early stage. One defining molecular feature of ancient DNA is the accumulation of uracil residues through postmortem cytosine deamination. Here, we leverage a uracil-DNA-specific labeling assay, originally developed for modern DNA, to evaluate whether extracts contain ancient DNA molecules. The assay, which requires only two days to complete, quantifies uracil residues using a uracil-DNA-binding protein and antibody-based labeling. After demonstrating reliable immobilization of short DNA fragments, we applied the assay to twenty archaeological samples spanning diverse contexts. Extracts with the highest inferred uracil contents were more likely to contain ancient DNA, whereas the lowest-ranked extracts were consistently negative. This assay may facilitate early prioritization of promising extracts and deprioritization of samples unlikely to yield informative sequencing data.

## Highlights

• A uracil-specific labeling assay detects uracil-containing DNA in extracts
• The assay was tested on archaeological skeletal and sediment samples
• Inferred uracil content correlates with ancient DNA detected by sequencing
• The assay can facilitate rapid prioritization of extracts in ancient DNA research

## Graphical abstract

**Figure:**
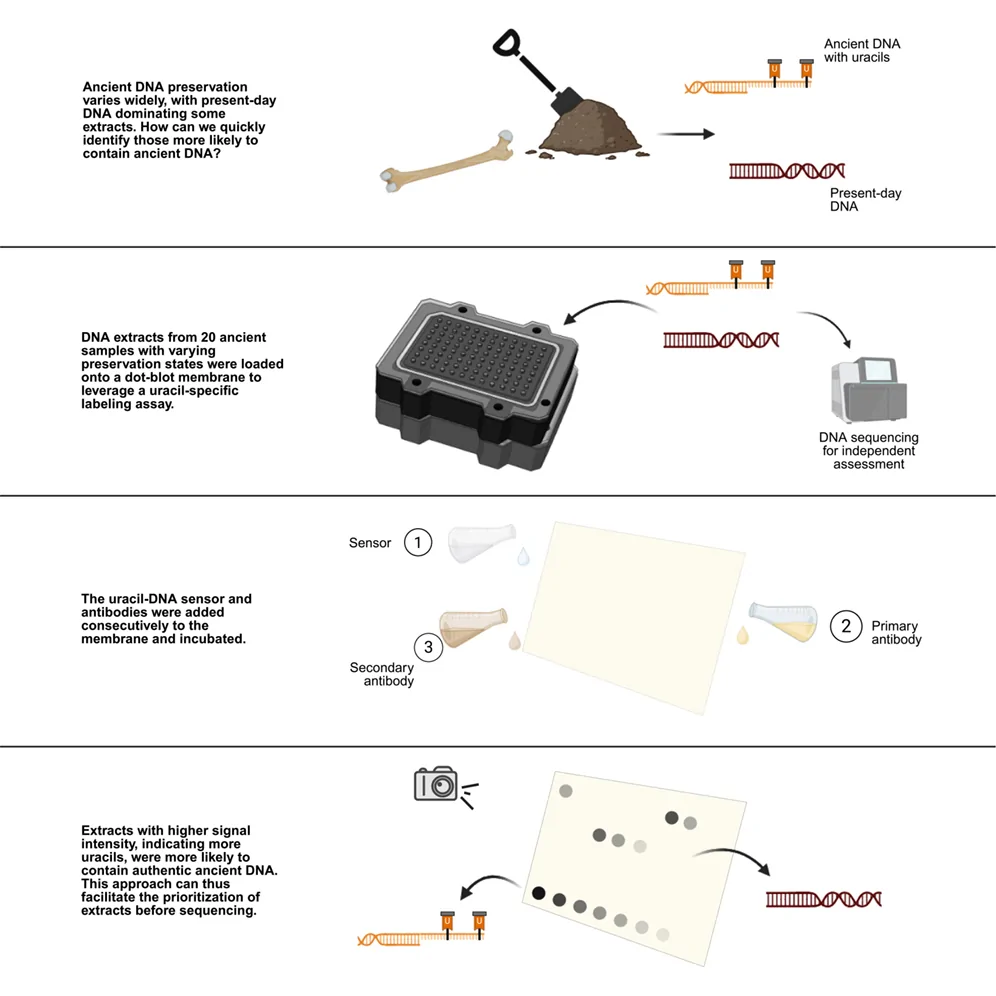

## Teaser

biological sciences; biochemistry; paleobiology; archeology

## Introduction

The analysis of ancient DNA (aDNA) has long been recognized as a powerful approach to study the past, with research over the last three decades yielding insights into the evolution of humans, animals, plants, and microorganisms. When applied to materials from archaeological contexts, aDNA analysis can provide direct insights into past human populations, domestication processes, ancient disease dynamics, and the composition of past ecosystems.^1^^,^^2^^,^^3^^,^^4^^,^^5^^,^^6^^,^^7^^,^^8^^,^^9^^,^^10^^,^^11^^,^^12^ ^and references therein^

A major limitation of current aDNA workflows is that they are both time- and resource-intensive. Laboratory protocols are typically tailored to recover highly fragmented and chemically damaged DNA molecules, requiring multi-step procedures and specialized reagents. As a result, several weeks or even months may pass between the arrival of a sample in the laboratory and the completion of an initial assessment of aDNA preservation. In addition, the associated costs with aDNA data generation are often substantial, although expenses can vary depending on the data-generation strategy chosen and on regional differences in reagent availability and pricing.^10^ These issues are particularly problematic when working with materials from contexts characterized by poor DNA preservation over time, either due to time depth, environmental conditions, or both.^13^^,^^14^^,^^15^ In addition, these challenges are often exacerbated in sedimentary aDNA (sedaDNA) studies, where DNA preservation can be highly heterogeneous, even within the same site or stratigraphic unit. This may be driven by variations in the source of the DNA, its depositional context, differences in micro-environmental conditions within the site, among other factors, many of which remain poorly understood.^16^^,^^17^^,^^18^^,^^19^ ^and references therein^ As such, considerable time, effort, and resources may be invested in samples from which ultimately little or no authentic aDNA can be recovered.

Over the years, different strategies have been developed to improve the efficiency of sample screening and reduce turnaround time and costs. One approach is to *a priori* select samples more likely to yield aDNA. Comparative analyses of aDNA yields among skeletal and dental remains have allowed for more targeted sampling strategies,^20^^,^^21^^,^^22^^,^^23^^,^^24^^,^^25^^,^^26^ and some studies have started to identify more favorable contexts for sediment sampling at archaeological sites.^27^^,^^28^^,^^29^^,^^30^ In addition, pre-screening of skeletal remains has been proposed through the evaluation of amino acid racemization, histological survey of bone micro-structure, quantification of collagen content, or the assessment of bone mineral structure and diagenetic alteration by spectroscopy (e.g., Fourier transform infrared [FTIR] and microRaman), all of which have been suggested to correlate, at least to some extent, with aDNA preservation.^31^^,^^32^^,^^33^^,^^34^^,^^35^^,^^36^^,^^37^^,^^38^^,^^39^^,^^40^^,^^41^ Alternatively, rapid assessment of aDNA preservation in the field may facilitate early prioritization of samples for more in-depth laboratory processing.^42^^,^^43^^,^^44^^,^^45^ Other approaches have focused on streamlining laboratory procedures for aDNA data generation. These include the automation of sample preparation protocols, which shortens hands-on preparation time and enables parallel processing of numerous samples^46^^,^^47^^,^^48^^,^^49^^,^^50^; the simplification of protocols to reduce steps and DNA loss^51^^,^^52^; the use of plate formats instead of single tubes to facilitate parallelization^53^; the substitution of particularly expensive reagents with more affordable alternatives to reduce costs^46^^,^^54^; and the pooling of either extracts or libraries for targeted enrichment and/or sequencing.^50^^,^^55^^,^^56^^,^^57^^,^^58^ Yet, there is still a need for rapid, cost-effective approaches that can assess the preservation of authentic aDNA in samples as early as possible in the process, prior to the investment of substantial time and costs.

The hallmark signature of aDNA degradation over time is cytosine deamination, resulting in uracil-containing DNA fragments.^59^^,^^60^^,^^61^ The conversion of cytosine bases to uracil residues, recognizable in sequencing data as cytosine-to-thymine (C-to-T) substitutions compared with a reference genome (or complementary guanine-to-adenine substitutions in double-stranded DNA libraries), tends to accumulate toward the ends of DNA fragments, in either single-stranded contexts or double-stranded contexts,^59^^,^^62^ although cytosine deamination can also occur in the internal parts of the DNA fragments, albeit in lower proportions.^63^^,^^64^ Unlike in aDNA, the deamination of cytosine bases to uracil residues is much less frequent in present-day DNA.^64^^,^^65^ Therefore, the presence of terminal C-to-T substitutions is routinely used in the authentication of aDNA fragments and their *in silico* separation from modern contaminating DNA.^64^^,^^66^^,^^67^^,^^68^^,^^69^ The occurrence of uracil residues in aDNA fragments has also been exploited to physically separate aDNA fragments from present-day DNA during library preparation.^70^^,^^71^^,^^72^ Here, we leverage an existing uracil-specific labeling assay originally developed to quantify the uracil content in modern DNA^73^ and test whether it could be applicable in the context of aDNA detection.

The uracil-specific labeling assay employs a uracil-DNA sensor protein engineered from the naturally evolved uracil-DNA glycosylase (UNG). The sensor is equipped with 3× FLAG-tag and carries two point mutations (D154N and H277N) that abolish the glycosylase activity by interfering with activation of the catalytic water molecule and leaving group departure,^74^ while preserving high-affinity uracil-DNA binding, as shown previously.^75^ This sensor has previously been used to quantify uracil content of human genomic DNA using a dot-blot assay, for *in situ* detection via immunocytochemistry, and for genome-wide profiling in a DNA immunoprecipitation sequencing approach termed U-DNA-seq.^76^ As an alternative, another uracil-DNA glycosylase, namely UdgX, has been used in a similar dot blot application,^77^ also in genome-wide^78^ and *in situ*^79^ analyses. In contrast, alternative methods typically rely on enzymatic processing of uracil-DNA followed by detection of abasic sites,^80^ which represents a more indirect readout and may be less specific when applied to potentially damaged aDNA samples. Direct quantification of uracil in DNA using the sensor protein in a dot-blot format requires only standard molecular biology laboratory equipment. The sensor can be produced recombinantly in an *ung-* *E. coli* strain (BL21(DE3) ung-151), and, in combination with commercially available antibodies (monoclonal anti-FLAG and anti-mouse-HRP), the experimental setup enables sensitive detection of uracil in DNA samples immobilized on a nylon membrane within 2 days^73^^,^^76^

In the current proof-of-concept study, we evaluate whether the uracil-DNA-specific labeling assay can serve as a rapid screening tool to identify DNA extracts more likely to contain authentic aDNA. Because postmortem cytosine deamination leads to elevated levels of uracil residues in aDNA compared with modern DNA, the relative abundance of uracil-containing DNA fragments may provide a simple means of distinguishing extracts containing ancient molecules from those dominated by modern DNA. We first assess the feasibility of implementing the method on short DNA fragments, as those typical of aDNA datasets, and then apply it to extracts from archaeological samples spanning diverse contexts and preservation conditions.

## Results

### Feasibility test for short DNA fragments

We first performed a feasibility test for the application of short DNA fragments on the membrane to be used in uracil-DNA determination. This control was necessitated by the characteristically short DNA fragments found in aDNA samples.^65^^,^^67^ The comparative experiment shows that the method can be applied to short fragments, like the ones expected in aDNA. In Figure 1, we show that a short oligonucleotide (25-base long) can be immobilized just as well as a 3 kb plasmid or as *E. coli* genomic DNA (gDNA; approximately 5 million base pairs). This result is a prerequisite for further analyses of short-length DNA fragments typical of aDNA, as it demonstrates the applicability of the uracil-DNA dot-blot method.

**Figure 1:**
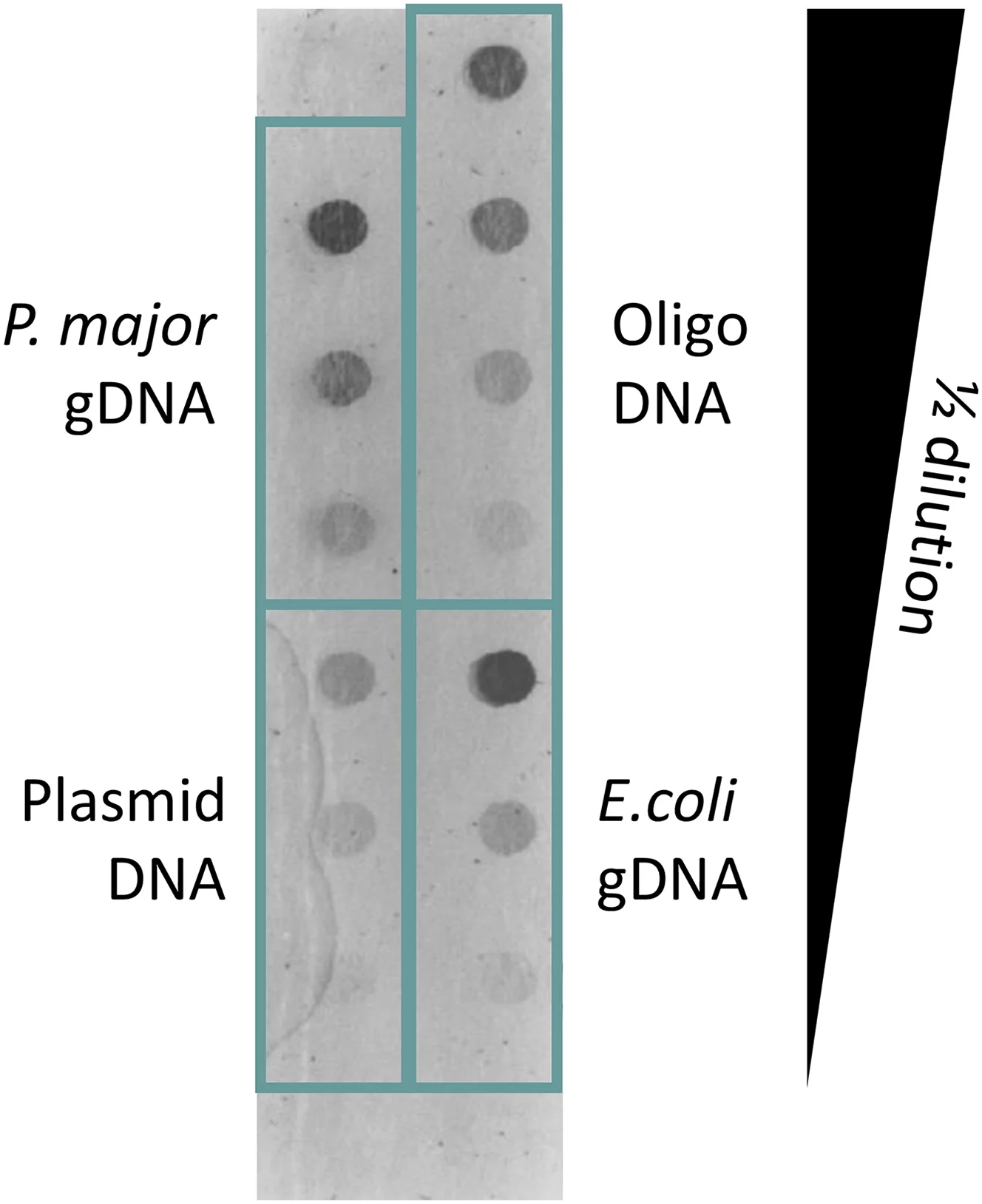
Immobilization of DNA fragments of varying sizes on a nylon membrane Samples were applied as a 1:2 dilution series (400, 200, and 100 ng DNA). An 800 ng oligonucleotide spot was included as an additional control. The membrane was stained with methylene blue.

### Dot-blot assay with ancient samples

Having demonstrated the feasibility of applying the procedure to short DNA fragments, we then proceeded to test the assay on DNA extracts generated from ancient samples. For this proof-of-concept study, we selected 20 samples from material that had previously been screened in our laboratory for aDNA preservation as part of different projects. Prior screening results allowed us to deliberately include samples representing diverse sample types, archaeological contexts, and levels of aDNA preservation. Specifically, the dataset comprised both sediment samples and skeletal and dental remains of animals, dated from the Middle Pleistocene to the Holocene, recovered from caves and a rock shelter in Europe and the southern Levant (Table 1).

**Table 1:** An overview of archaeological samples included in the study and their aDNA preservation status, as determined using sequencing data

Sample abbrv.	Type	Chronostratigraphy	Site	Type of site	Ref. for the site/layer	Ancient mammalian mtDNA	Ancient microbial DNA
S1	sediment	Upper Pleistocene?	Perspektywiczna Cave, Poland	cave (near the entrance)	Krajcarz et al.^81^	–	–
S2	sediment	Upper Pleistocene?				+	–
S3	sediment	Lower Holocene				+	–
S4	sediment	Lower Holocene				–	–
S5	sediment	Holocene				–	–
S6	sediment	Holocene				++	–
S7	sediment	Holocene				+	–
S8	sediment	Upper Pleistocene (Mousterian)	Sefunim Cave, Israel	cave	Slon et al.^29^	–	–
S9	sediment	Upper Pleistocene (Mousterian)				–	+
S10	sediment	Upper Pleistocene (Mousterian)	Cueva Millán, Spain	rock shelter	Sánchez-Yustos et al.^82^	–	–
S11	sediment	Upper Pleistocene (initial Upper Paleolithic)				+	–
S12	sediment	Upper Pleistocene (initial Upper Paleolithic)				–	–
S13	sediment	Upper Pleistocene (initial Upper Paleolithic)				++	–
S14	sediment	Middle Pleistocene	Biśnik Cave, Poland	cave	Krajcarz et al.^83^	++	++
S15	sediment	Middle Pleistocene				++	++
S16	sediment	Middle Pleistocene				–	–
AT1	animal tooth	Upper Pleistocene (Mousterian)	Sefunim Cave, Israel	cave	Slon et al.^29^	–	–
AT2	animal tooth	Upper Pleistocene (Late Upper Paleolithic)				–	–
AB1	animal bone	Upper Pleistocene (Upper Paleolithic, Aurignacian)	Istállóskő Cave, Hungary	cave	Lengyel et al.^84^	++	–
AB2	animal bone	Upper Pleistocene (Upper Paleolithic, Aurignacian)				++	–

Preservation of aDNA was defined based on elevated terminal C-to-T substitutions compared to a relevant reference genome; see details in Tables S3 and S4 for the mammalian mtDNA data and the shotgun data, respectively. “++” indicates significant elevation of C-to-T substitutions at both sequence ends (based on the lower bound of a 95% binomial CI at least 9.5%), consistent with authentic aDNA; “+” indicates significant elevation of C-to-T substitutions at one sequence end only, which may reflect a lower extent of damage or limited statistical power; “−” indicates no significant C-to-T substitutions at either end, indicating no evidence for authentic aDNA in the data. Note that the assessment of aDNA preservation status shown here is independent from the dot-blot assay. For full sample context, see Table S1.

Screening for aDNA preservation had first been performed by targeted enrichment of the DNA libraries for mammalian mtDNA.^49^ Taxonomic assignment of sequenced DNA fragments was made at the biological family level. Then, for each family identified in the data, the DNA fragments were evaluated for the presence of terminal C-to-T substitutions to determine their ancientness.^85^ On the basis of these prior analyses, half of the samples that we chose (8 sediment samples and two animal bones) were known to preserve traces of ancient mammalian mtDNA (Figure S2; Table S3).

Later, as part of the current study, we performed shotgun sequencing of all samples to assess whether ancient microbial DNA was present in our datasets. After mapping to a concatenated reference genome comprising 44 microbial genomes (Table S2), authentication of the ancient origin of the recovered DNA fragments again relied on elevated terminal C-to-T substitutions.^64^ Three samples were found to contain ancient microbial DNA, two of which had previously yielded ancient mammalian mtDNA, while the third had not (Figure S2; Table S4).

To evaluate whether the UNG sensor-based method for quantifying uracil content can be used to detect the presence of aDNA, we performed a dot-blot assay (test 1) on a set of samples previously characterized through mitochondrial capture and sequencing. This set included two sediment samples and two animal bone samples that were known to contain ancient mammalian mtDNA (detailed information on samples can be found in Tables 1 and S1). Using the dot-blot assay, uracil-containing DNA fragments were detected in all four extracts, with point estimates of the frequencies ranging between 733 and 2,681 uracil residues per million bases. In comparison, estimates of uracil content in modern DNA tend to be much lower, ranging from approximately 1–10 uracil residues per million DNA bases in human cells to approximately 3–6 uracil residues per million bases in wild-type *E. coli* and ∼15 uracil residues per million bases in untreated *Drosophila* S2 cells.^73^^,^^86^ We do note that estimates of uracil content in modern DNA reported in the literature can vary substantially among different biological substrates and between measurement systems (e.g., see Olinski et al.^87^ and references therein). Overall, our results here show that extracts with known aDNA preservation produce a detectable and consistent signal in the assay (Figure 2A; Table S5).

**Figure 2:**
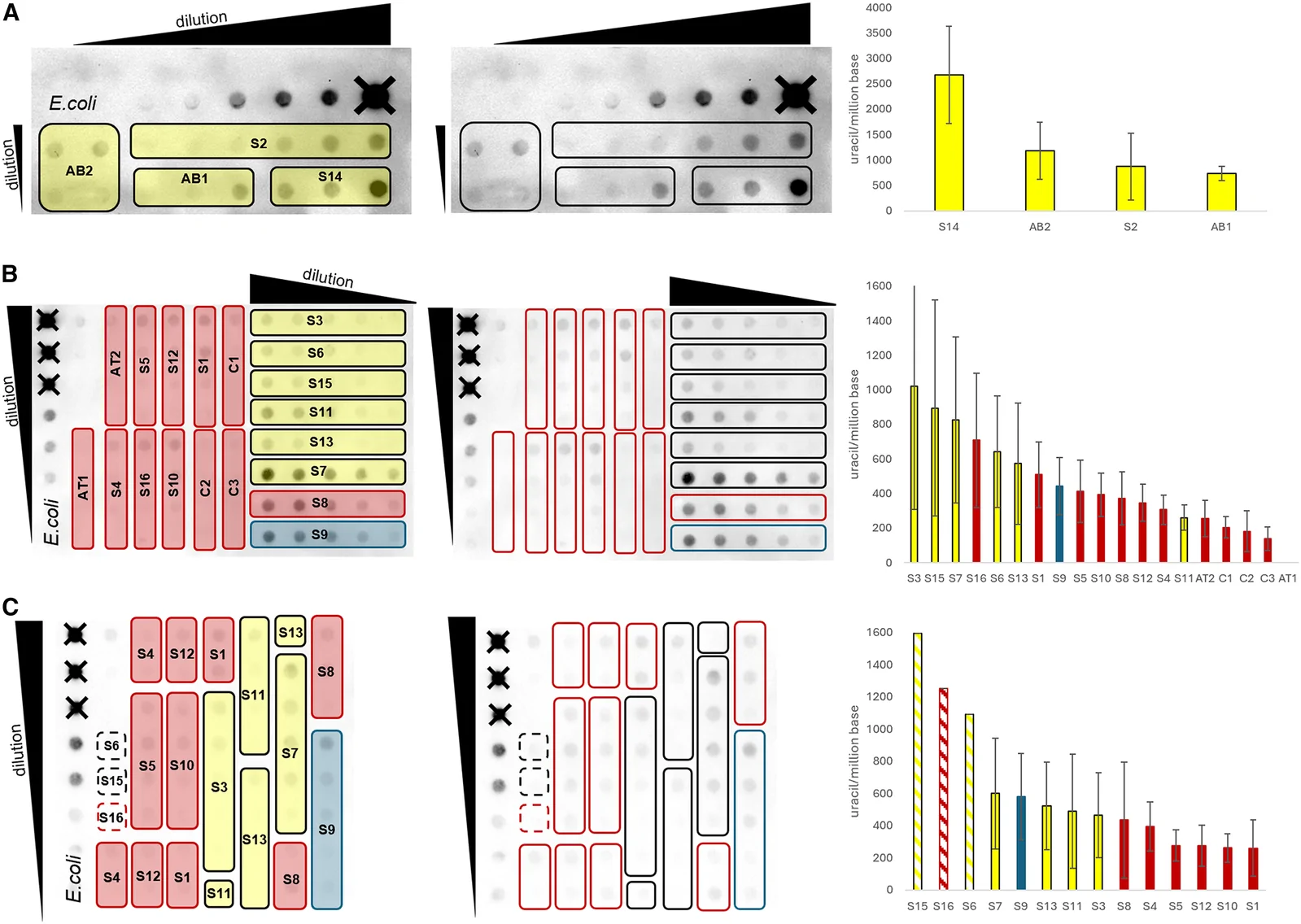
Images of the dot-blot membranes and the calculated amount of uracil residues per sample (A–C) For all tests, the membrane setup is shown on the left panels; for sample ID details, see Tables 1 and S1. Crossed-out wells indicate over-saturated points that were not taken into account in the standard curves. In all images, yellow represents a sample positive for ancient mammalian mtDNA; blue represents a sample positive for ancient microbial DNA but negative for ancient mammalian mtDNA; and red represents a sample negative for both ancient mammalian mtDNA and ancient microbial DNA. *E. coli* dilutions (as standard) are shown in the first columns. Uracil amounts, calculated using a linear fit, are shown on the right panels. Data are shown as the mean count of uracil residues per million bases ± SD. (A) The results of test 1 containing four aDNA-positive samples with different numbers of dilutions (image taken with an exposure time of 162.6 s). (B) The results of test 2, containing both aDNA-positive and aDNA-negative samples, as well as extraction negative controls (image taken with an exposure time of 26.3 s). (C) The results of test 3, containing both aDNA-positive and aDNA-negative samples previously included as part of test 2 (image taken with an exposure time of 13.1 s). Dashed columns indicate samples without dilution.

We then repeated the experiment using a larger and more diverse set of samples (test 2), this time including both aDNA-positive and aDNA-negative extracts (Tables 1 and S1). Extracts classified as aDNA-positive exhibited uracil frequencies with point estimates ranging from 261 to 1,023 per million bases, whereas samples with no evidence for aDNA preservation had values ranging from 0 to 708 uracil residues per million bases. We note that extraction negative controls yielded uracil signal levels within the range observed for aDNA-negative samples (Table S5). When ranked according to their inferred uracil content, four of the five extracts with the highest uracil frequencies corresponded to samples independently identified as containing aDNA based on sequencing results, whereas the five extracts with the lowest frequencies all originated from samples or from negative controls in which no evidence of aDNA was detected in the sequencing data (Figure 2B).

To assess the reproducibility of the experiment when applied to ancient samples, we performed a third dot-blot assay (test 3), in which we re-ran the remaining available extracts from test 2. Point estimates for the uracil content of the extracts ranged between 465 and 1,595 uracil residues per million bases for the aDNA-positive samples and between 260 and 1,252 for aDNA-negative ones. We note that the three extracts with the highest uracil values here were measured only once, rather than in serial dilution, due to limited extract availability, which may have affected the precision of these measurements (Table S5). Overall, although absolute uracil counts per sample differed from those observed in test 2, the ranking pattern was largely maintained. When considering the five highest-ranking extracts, three corresponded to aDNA-positive samples, as identified by sequencing. When excluding the three single-measurement extracts, all the next five highest-ranking samples indeed contained aDNA. Finally, as in test 2, the five extracts with the lowest inferred uracil frequencies were all derived from samples for which no evidence of aDNA preservation was detectable in the sequencing data (Figure 2C).

### Association between inferred uracil content and sequencing results

We first incorporated the results of the dot-blot assays into a logistic regression model to evaluate the ability of inferred uracil counts to predict the aDNA preservation status of extracts. When taking naively the per-sample average of all dot-blot assays, we found that the uracil content strongly predicts the samples with significant presence of aDNA in the mammalian mtDNA capture (likelihood ratio test *p* value = 0.00013), or with significant presence of aDNA either in the mammalian mtDNA capture data or in microbial reads from shotgun sequencing (likelihood ratio test *p* value = 0.00016). To better account for potential batch effects introduced by different membranes and to avoid pseudo-replication, we modeled them explicitly by regressing the measurements obtained in test 2 and test 3 to obtain a single uracil-content estimate and excluded test 1 from this analysis, as it does not share any sample with the other experiments. Even using this more conservative approach, we find that the measurements of the dot-blot assay significantly predict the presence of aDNA in the mammalian mtDNA capture (likelihood ratio test *p* value = 0.00526), as well as in either the mammalian mtDNA capture data or the microbial DNA data generated via shotgun sequencing (likelihood ratio test *p* value = 0.00544).

We then tested whether uracil content predicts the number of deaminated fragments recovered in the mammalian mtDNA capture data, i.e., the fragments that could be used for downstream analyses. For this, we used a negative binomial model with total read count as an offset to account for sequencing depth and overdispersion, thus providing a measure of aDNA yield in terms of usable deaminated fragments per extract. Uracil content was a significant positive predictor of deaminated fragments yield (β = 0.005, z = 2.72, *p* value = 0.007), with a 0.5% increase in deaminated fragment count for each added uracil per million bases, after accounting for sequencing depth (Figure S3). For the samples reported here, an increase in one standard deviation of uracil content (272.36 uracil residues per million bases) corresponded to a 3.8-fold increase in the yield of sequenced deaminated DNA fragments. Note that while a negative binomial model was supported over a zero-inflated model (see STAR Methods), the latter also showed a significant relationship between uracil and yield in terms of deaminated fragments (*p* value = 0.0154).

Lastly, we explored whether the number of fragments attributed to taxa authenticated as ancient based on the deamination frequency, per mg of input material, is indeed more abundant in sediment samples with a higher inferred uracil content. As expected from the previous tests, the two correlated positively when using all data (Spearman’s ρ = 0.87, *p* value = 0.00013; Figure S4). This association remained positive but was no longer statistically significant when samples yielding no authenticated aDNA were excluded (Spearman’s ρ = 0.71, *p* value = 0.14), likely reflecting the small number of positive samples.

## Discussion

As a screening tool for aDNA, the principal advantage of the uracil-DNA dot-blot assay is that it introduces an early decision point into a library-based aDNA workflow, allowing DNA extracts to be evaluated before substantial laboratory time, effort, and resources are invested in downstream procedures. The dot-blot assay requires approximately $3–6 in consumable reagents per sample, can be completed within 1–2 laboratory days, and allows the simultaneous processing of 10–20 samples per membrane (Table S6). In comparison, proceeding directly to DNA library preparation followed by low-level sequencing typically requires approximately $45–150 per sample in consumables and 3–6 hands-on laboratory days, with the number of samples that can be processed in parallel differing considerably between manual preparation and automated liquid handling protocols. Workflows including targeted enrichment require an additional $50–250 per sample and can double the hands-on processing time (Table S6). We note that these estimates should be regarded as indicative only, as consumable costs, laboratory processing times, and sample throughput vary substantially among laboratories. Moreover, in practice, laboratory processing represents only part of the overall project timeline, with additional time often arising from sample batching, laboratory scheduling and workload, instrument availability, and access to external sequencing facilities where applicable. Consequently, the actual time between sample acquisition and first sequencing results often extends to weeks or months, if not more. The ability to prioritize extracts likely containing aDNA fragments, or, perhaps more importantly, to deprioritize extracts unlikely to contain aDNA, before embarking on these downstream laboratory procedures, therefore constitutes the main practical value of applying this assay within an aDNA workflow.

In its current configuration, the main drawback of the dot-blot assay as a screening tool for aDNA is its lack of specificity, as the method is not designed to distinguish the source of the uracil-containing DNA fragments. Indeed, a positive signal could be derived from aDNA endogenous to the sample but also from exogenous contamination with ancient bacterial or fungal DNA. Moreover, the assay is not perfectly predictive. As such, a high-ranking extract, in terms of inferred uracil content, may still not contain aDNA of interest to the research team, or even any aDNA at all, at least within the limits of detection with the sequencing and data analysis schemes employed here. Such false-positive cases may lead to unnecessary investment of laboratory time, effort, and resources in subsequent library preparation, sequencing, and data analysis, without ultimately yielding aDNA data applicable for the intended research question.

A second limitation is that the authentication of aDNA and its suitability for downstream analyses are not equivalent. While the presence of postmortem damage is a prerequisite for establishing the authenticity of aDNA, it does not necessarily imply that sufficient endogenous DNA has been preserved for meaningful downstream analyses, such as genome reconstruction or population-genetic studies. This distinction is particularly relevant for substrates in which DNA preservation is inherently challenging, such as archaeological sediments. At the same time, our data suggest that false negatives may be less common. Extracts with the lowest inferred uracil contents were consistently negative in the sequencing data, whereas samples with intermediate uracil contents included both positive and negative samples. On the basis of the samples tested here, we do not provide a threshold based on inferred uracil counts to distinguish positive samples from negative samples. Thus, rather than providing a definitive classification, the assay may best be viewed as a prioritization tool. In practice, the most appropriate threshold will depend on the intended downstream application and the objectives and scope of a given project. For example, researchers may choose to prioritize a certain fraction of the highest-ranking extracts for downstream analyses, while recognizing that additional sequencing-based analyses remain necessary to follow-up on potentially positive samples and determine whether sufficient endogenous DNA has been preserved for a particular research objective. In other contexts, the dot-blot assay could be used rather to deprioritize extracts with the lowest inferred uracil content, as these are the least likely to contain aDNA.

Third, the dot-blot assay requires the use of an aliquot of the DNA extract, which, unlike a DNA library, represents a finite resource. Here, depending on the starting DNA concentration, between 2.6 and 32.3 μL of extract was required for the assay, corresponding to 5.2%–64.6% of the original extract volume (Table S5). In comparison, we used 5 or 10 μL of extract as input into DNA library preparation (10%–20% of the original extract volume; Table S1). For some samples, where repeated library preparations would be needed, dedicating a portion of the extract to the dot-blot assay may constrain subsequent analyses. One potential mitigation strategy would be to increase the elution volume during DNA extraction (e.g., to 100 μL), thereby reducing the proportion of the extract required for a rapid screening through the dot-blot assay.

Lastly, a minor drawback is that although the assay itself is simple and does not entail high-cost instrumentation, signal detection requires a chemiluminescence imaging system. Such equipment may not be routinely available in dedicated aDNA laboratories and may, therefore, necessitate collaboration with other facilities for integration into an aDNA workflow.

Overall, in this study, we evaluated whether a dot-blot assay, developed previously to detect and estimate the abundance of uracil residues in modern DNA,^73^ could be repurposed as a rapid tool to screen extracts made from archaeological samples for the presence of aDNA. The ability to assess aDNA preservation at the extract stage (i.e., prior to the time- and cost-consuming steps of library preparation, targeted enrichment and sequencing) could be of particular interest in the context of aDNA research conducted in regions and/or timescales where poor DNA preservation is expected, or for samples from which aDNA yields tend to be low, such as sediment samples.

We first verified, using an artificial oligonucleotide, that short DNA fragments of the size expected in ancient samples can successfully be immobilized onto the membrane used in the dot-blot assay. We then demonstrated, using a restricted set of samples, that DNA extracts derived from archaeological bones and sediments indeed bind efficiently to the membrane and generate a measurable signal in the dot-blot assay, from which uracil content can be inferred. Lastly, when applied to a larger set of archaeological samples from different provenances, we showed that ranking according to the inferred content of uracil residues can be predictive of the presence of deaminated DNA fragments. Specifically, among the highest-ranked samples in terms of inferred uracil content, most were confirmed to contain aDNA by sequencing, whereas the lowest-ranked samples were consistently negative for aDNA.

Our findings support the utility of uracil quantification as an indicator of aDNA preservation in archaeological samples. We therefore propose that the dot-blot assay can serve as a practical tool to prioritize large-scale screening efforts, either by selecting high-ranking extracts for further processing and/or by excluding low-ranking ones unlikely to eventually yield informative aDNA data.

### Limitations of the study

The principal limitation of this study is the relatively small number of archaeological samples included (*n* = 20). Although intentionally designed as a proof-of-concept, and while we strove to span different sample types and archaeological contexts, this dataset is limited in its ability to establish widely applicable decision thresholds or to comprehensively evaluate the performance of the assay across different depositional environments and preservation conditions. Future studies including larger number of samples from a broader range of archaeological contexts will be needed to refine threshold values relevant to different research objectives and strategies, assess the generality of the observed patterns, and further evaluate the applicability of the assay across different substrates and research questions.

## Resource availability

### Lead contact

Requests for further information and resources and reagents should be directed to and will be fulfilled by the lead contact, Viviane Slon (viviane@tauex.tau.ac.il).

### Materials availability

Requests for materials related to the dot-blot assays should be addressed to Beáta G. Vértessy (vertessy.beata@ttk.hu).

### Data and code availability

• All sequencing data generated for this study have been deposited in the European Nucleotide Archive (ENA): PRJEB112136. We note that further analyses of these genetic data are ongoing, and, therefore, we kindly ask that researchers intending to use them contact the corresponding authors prior to publication.
• This paper does not report original code.
• Any additional information required to reanalyze the data reported in this paper is available from the lead contact upon request.

## Acknowledgments

This work was funded by the John Templeton Foundation (grant #62571 to S.S. and V.S.) and supported by the National Research, Development and Innovation Office of Hungary (K146890, 2025–1.2.7-HU-CN-PARTNER-2025–00025 to B.G.V.) and the ICGEB Research Grants Programme 2023 (CRP/HUN23-02 to B.G.V.). aDNA research at Perspektywiczna Cave is funded by the United States-Israel Binational Science Foundation (grant 2021219 to V.S.) and at Sefunim Cave by the Israel Science Foundation (grant #2075/22 to V.S.). Research at Cueva Millán is conducted within the framework of the PIONEERS project (PID2024-162134NB-I00 to M.Á.R.-G. and P.S.-Y.), funded by MICIU/AEI/10.13039/501100011033. G.L. was supported by Narodowe Centrum Nauki, Poland (grant number UMO-2019/33/B/HS3/02264). We thank Giulia Di Crosta, Dina Gerbek, and Prajjval Pratap Singh for help in the aDNA lab; the team of the DNA Sequencing Unit at the G.S. Wise Faculty of Life Sciences (TAU) for assistance with shotgun sequencing; Amit Mualem and Chen Zeigen for assistance with sediment sampling; and Jamie Clark for selecting animal teeth from Sefunim Cave. We thank the three reviewers for their input and our colleagues from other aDNA facilities for kindly sharing information about cost calculations and sample processing times in their laboratories.

## Author contributions

N.D.D., B.G.V., and V.S. designed the experiments; N.D.D., D.C., A.J., A.L., V.V., and V.S. generated genetic data; N.D.D., F.M., D.C., V.V., and V.S. analyzed the genetic data; N.D.D., V.P.-S., and E.H. carried out the dot-blot experiments and analyzed the results under the supervision of A.B. and B.G.V.; K.C., A.W.K., M.T.K., M.K., G.L., M.Á.R.-G., P.S.-Y., R.S., M.S.-P., and J.W. contributed samples and archaeological expertise; S.S., B.G.V., and V.S. acquired funding for the project; N.D.D., V.P.-S., F.M., A.B., B.G.V., and V.S. wrote the manuscript with input from all authors.

## Declaration of interests

The authors declare no competing interests.

## STAR★Methods

### Key resources table

**Table:** 

REAGENT or RESOURCE	SOURCE	IDENTIFIER
**Antibodies**		

ANTI-FLAG M2 antibody, mouse monoclonal	Sigma-Aldrich	Cat#F3165; RRID: AB_259529
Anti-Mouse IgG-peroxidase antibody produced in rabbit	Sigma-Aldrich	Cat#A9044; RRID: AB_258431

**Bacterial and virus strains**		

*Escherichia coli* BL21 (DE3) ung-151 strain	Lari et al.^88^	N/A

**Biological samples**		

Sediment sample 1	This study	S1
Sediment sample 2	This study	S2
Sediment sample 3	This study	S3
Sediment sample 4	This study	S4
Sediment sample 5	This study	S5
Sediment sample 6	This study	S6
Sediment sample 7	This study	S7
Sediment sample 8	This study	S8
Sediment sample 9	This study	S9
Sediment sample 10	This study	S10
Sediment sample 11	This study	S11
Sediment sample 12	This study	S12
Sediment sample 13	This study	S13
Sediment sample 14	This study	S14
Sediment sample 15	This study	S15
Sediment sample 16	This study	S16
Animal tooth sample 1	This study	AT1
Animal tooth sample 2	This study	AT2
Animal bone sample 1	This study	AB1
Animal bone sample 2	This study	AB2
*Escherichia coli* CJ236 genomic DNA	Laboratory stock	N/A

**Chemicals, peptides, and recombinant proteins**		

0.5 M EDTA sln, pH 8	Fisher	Cat#BP2482
1 M Tris-HCl sln, pH 8	Bio-Lab	Cat#208523
Proteinase K (kit component)	Roche	Cat#5114403001
Guanidine hydrochloride	Sigma-Aldrich	Cat#G3272
2-Propanol	Merck	Cat#109634
3 M Sodium acetate sln, pH 5.2	Sigma-Aldrich	Cat#S7899
Buffer PE	Qiagen	Cat#19065
Tween 20	Bio-Lab	Cat#20452335
5 M NaCl sln	Sigma-Aldrich	Cat#59222C
20X SSC buffer	Thermo Fisher Sci.	Cat#AM9770
20% (wt/vol) SDS sln	Bio-Lab	Cat#19812375
T4 RNA ligase reaction buffer	NEB	Cat#B0216L
ATP solution 100 mM	Thermo Fisher Sci.	Cat#R0441
Klenow fragment, including 10X rxn buffer	Thermo Fisher Sci.	Cat#EP0052
T4 polynucleotide kinase 10 U/μL	Thermo Fisher Sci.	Cat#EK0031
FastAP thermosensitive alkaline phosphatase	Thermo Fisher Sci.	Cat#EF0654
T4 DNA ligase, HC 30 U/μL	Thermo Fisher Sci.	Cat#EL0013
T4 DNA ligase, 5 U/μL	Thermo Fisher Sci.	Cat#EL0011
Dynabeads MyOne Streptavidin C1	Life Technologies	Cat#65001
dNTP (25 mM each)	Thermo Fisher Sci.	Cat#R1121
pUC 19 vector	NEB	Cat#N3041S
Fast SYBR Green Master Mix	Thermo Fisher Sci.	Cat#4385612
AccuPrime Pfx DNA polymerase	Thermo Fisher Sci.	Cat#12344024
Herculase II DNA polymerase, including 5X rxn buffer	Agilent	Cat#600679
1 M Sodium hydroxide	Fisher Scientific	Cat#S25549A
Capture baits	Slon et al.^89^	N/A
Blocker 1 (500 μM)	Slon et al.^49^	Sigma-Aldrich
Blocker 2 (500 μM)	Slon et al.^49^	Sigma-Aldrich
Blocker 3 (500 μM)	Slon et al.^49^	Sigma-Aldrich
Blocker 4 (500 μM)	Slon et al.^49^	Sigma-Aldrich
2x HI-RPM hybridization buffer	Agilent	Cat#5188–5220
Agilent blocking agent	Agilent	Cat#5188–5220
GeneAmp 10X PCR Buffer II	Thermo Fisher Sci.	Cat#N8080010
Sera-Mag Speedbeads	Cytiva	Cat#66152104010150
1 M KCl	Sigma-Aldrich	Cat#60142
Amersham Hybond-N+ membrane	Cytiva	Cat#RPN1210B
Skim milk powder	Sigma-Aldrich	Cat#70166
Ultrapure salmon sperm DNA	Thermo Fisher Sci.	Cat#15632011
2-mercaptoethanol	Sigma-Aldrich	Cat#8057400005
ECL reagents	Millipore	Cat#WBULP
3x-FLAG-ΔUNG sensor	Róna et al.^73^	N/A

**Critical commercial assays**		

GeneJET Genomic DNA Purification kit	Thermo Fisher Sci.	Cat#K0721
High Pure Viral Nucleic Acid Large Volume Kit	Roche	Cat#5114403001
MinElute PCR Purification Kit	Qiagen	Cat#28006
NucleoSpin Plasmid Mini kit	Macherey-Nagel	Cat#740588.250
Quick-DNA Miniprep Plus kit	Zymo Research	Cat#D4068
Qubit dsDNA HS Assay Kit	Thermo Fisher Sci.	Cat#Q32851
Qubit RNA BR Assay Kit	Thermo Fisher Sci.	Cat#Q10210

**Deposited data**		

Sequencing data	This study	ENA: PRJEB112136
*Yersinia_similis* genome assembly	Friedrich-Loeffler-Institut	https://www.ncbi.nlm.nih.gov/datasets/genome/GCF_000582515.1/
*Yersinia_frederiksenii* genome assembly	Los Alamos National Laboratory	https://www.ncbi.nlm.nih.gov/datasets/genome/GCF_000834215.1/
*Yersinia_intermedia* genome assembly	Los Alamos National Laboratory	https://www.ncbi.nlm.nih.gov/datasets/genome/GCF_000834515.1/
*Yersinia_aldovae* genome assembly	Naval Medical Research Center	https://www.ncbi.nlm.nih.gov/datasets/genome/GCF_000173735.1/
*Yersinia kristensenii* genome assembly	State Research Center for Applied Microbio. & Biotech.	https://www.ncbi.nlm.nih.gov/datasets/genome/GCF_002794215.1/
*Yersinia enterocolitica* genome assembly	Sanger Institute	https://www.ncbi.nlm.nih.gov/datasets/genome/GCF_000009345.1/
*Aspergillus fumigatus* genome assembly	J. Craig Venter Institute	https://www.ncbi.nlm.nih.gov/datasets/genome/GCF_000002655.1/
*Yersinia enterocolitica* subsp. *palearctica Y11* genome assembly	Max von Pettenkofer-Institute	https://www.ncbi.nlm.nih.gov/datasets/genome/GCF_000253175.1/
*Mycobacterium leprae* genome assembly	Nat. JALMA Ins. for Leprosy & Other Mycobacterial Diseases	https://www.ncbi.nlm.nih.gov/datasets/genome/GCF_003253775.1/
*Pseudomonas putida* genome assembly	The Institute for Genomic Research	https://www.ncbi.nlm.nih.gov/datasets/genome/GCF_000007565.2/
*Acinetobacter baylyi* genome assembly	The French National Sequencing Center (Genoscope)	https://www.ncbi.nlm.nih.gov/datasets/genome/GCF_000046845.1/
*Sphingomonas paucimobilis* genome assembly	AIMST University	https://www.ncbi.nlm.nih.gov/datasets/genome/GCF_003314795.2/
*Stenotrophomonas maltophilia* genome assembly	Wellcome Trust Sanger Institute	https://www.ncbi.nlm.nih.gov/datasets/genome/GCF_000072485.1/
*Shewanella oneidensis* genome assembly	The Institute for Genomic Research	https://www.ncbi.nlm.nih.gov/datasets/genome/GCF_000146165.2/
*Cupriavidus necator* genome assembly	Gottingen Genomics Laboratory	https://www.ncbi.nlm.nih.gov/datasets/genome/GCF_000009285.1/
*Bradyrhizobium diazoefficiens* genome assembly	Kazusa DNA Research Institute	https://www.ncbi.nlm.nih.gov/datasets/genome/GCF_000011365.1/
*Streptomyces coelicolor* genome assembly	Sanger Institute	https://www.ncbi.nlm.nih.gov/datasets/genome/GCF_000203835.1/
*Cutibacterium acnes* genome assembly	Goettingen Genomics Laboratory	https://www.ncbi.nlm.nih.gov/datasets/genome/GCF_000008345.1/
*Micrococcus luteus* genome assembly	US Food and Drug Administration	https://www.ncbi.nlm.nih.gov/datasets/genome/GCF_009730415.1/
*Bacillus subtilis str. 168* genome assembly	BSNR	https://www.ncbi.nlm.nih.gov/datasets/genome/GCF_000009045.1/
*Thermotoga petrophila* genome assembly	US DOE Joint Genome Institute	https://www.ncbi.nlm.nih.gov/datasets/genome/GCF_000016785.1/
*Mycobacterium tuberculosis* H37Rv genome assembly	Sanger Institute	https://www.ncbi.nlm.nih.gov/datasets/genome/GCF_000195955.2/
*Methilobacterium nodulans* genome assembly	US DOE Joint Genome Institute (JGI-PGF)	https://www.ncbi.nlm.nih.gov/datasets/genome/GCF_000022085.1/
*Gloeobacter violaceus* genome assembly	Kazusa DNA Research Institute	https://www.ncbi.nlm.nih.gov/datasets/genome/GCF_000011385.1/
Dictyoglomus thermophilum genome assembly	The J. Craig Venter Institute	https://www.ncbi.nlm.nih.gov/datasets/genome/GCF_000020965.1/
*Yersinia pestis chr* genome assembly	Parkhill et al.^90^	https://www.ncbi.nlm.nih.gov/nuccore/NC_003143.1/
*Yersinia pestis pMT1* genome assembly	Parkhill et al.^90^	https://www.ncbi.nlm.nih.gov/nuccore/NC_003134.1
*Yersinia pestis pPCP1* genome assembly	Parkhill et al.^90^	https://www.ncbi.nlm.nih.gov/nuccore/NC_003132.1
*Yersinia pestis pCD1 shared* genome assembly	Parkhill et al.^90^	https://www.ncbi.nlm.nih.gov/nuccore/NC_003131.1
*Yersinia pseudotuberculosis chr* genome assembly	Chain et al.^91^	https://www.ncbi.nlm.nih.gov/nuccore/NC_006155.1
*PhiX174* genome assembly	Sanger et al.^92^	https://www.ncbi.nlm.nih.gov/nuccore/NC_001422.1
*Broad host plasmid PB10* genome assembly	Schlüter et al.^93^	https://www.ncbi.nlm.nih.gov/nuccore/NC_004840.1
*Salmonella enterica* subsp. *enterica serovar* genome assembly	Deng et al.^94^	https://www.ncbi.nlm.nih.gov/nuccore/NC_004631.1
*Salmonella enterica2* genome assembly	Parkhill et al.^95^	https://www.ncbi.nlm.nih.gov/nuccore/NC_003198.1
*Mycobacterium tuberculosis_H37Rv* genome assembly	Cole et al.^96^	https://www.ncbi.nlm.nih.gov/nuccore/NC_000962.3
*Bacillus subtilis* genome assembly	Kunst et al.^97^	https://www.ncbi.nlm.nih.gov/nuccore/NC_000964.3/
*Clostridium perfringens* genome assembly	Shimizu et al.^98^	https://www.ncbi.nlm.nih.gov/nuccore/NC_003366.1
*Methanosphaerula palustris* genome assembly	Cadillo-Quiroz et al.^99^	https://www.ncbi.nlm.nih.gov/nuccore/NC_011832.1/
*Streptomyces coelicolor A3(2)* genome assembly	Bentley et al.^100^	https://www.ncbi.nlm.nih.gov/nuccore/NC_003888.3/
*Curibacterium acnes* genome assembly	Brüggemann et al.^101^	https://www.ncbi.nlm.nih.gov/nuccore/NC_006085.1/
*Escherichia coli K12* genome assembly	Blattner et al.^102^	https://www.ncbi.nlm.nih.gov/nuccore/NC_000913.3
*Corynebacterium glutamicum* genome assembly	Kalinowski et al.^103^	https://www.ncbi.nlm.nih.gov/nuccore/NC_003450.1/
*Nocardioides* sp. genome assembly	Coleman et al.^104^	https://www.ncbi.nlm.nih.gov/nuccore/NC_008699.1/
*Shewanella oneidensis MR-1* genome assembly	Heidelberg et al.^105^	https://www.ncbi.nlm.nih.gov/nuccore/NC_004347.1/

**Oligonucleotides**		

TL181 adapter	Gansauge et al.^46^	Sigma-Aldrich
TL159 splinter	Gansauge et al.^46^	Sigma-Aldrich
CL128 primer	Gansauge et al.^46^	Sigma-Aldrich
CL53 adapter	Gansauge et al.^46^	Sigma-Aldrich
TL178 adapter	Gansauge et al.^46^	Sigma-Aldrich
IS7 primer	Gansauge et al.^46^	Sigma-Aldrich
IS8 primer	Gansauge et al.^46^	Sigma-Aldrich
IS10 probe A	Gansauge et al.^46^	Sigma-Aldrich
CL107 reverse primer B	Gansauge et al.^46^	Sigma-Aldrich
CL118 probe	Gansauge et al.^46^	Sigma-Aldrich

**Software and algorithms**		

AdapterRemoval v. 2.3.2	Schubert et al.^106^	https://github.com/mikkelschubert/adapterremoval
nf-core/eager v. 2.4.7	Yates et al.^107^	https://nf-co.re/eager/2.4.7/
samtools v. 1.19.2	Li et al.^108^	https://github.com/samtools/samtools/releases/tag/1.19.2
BWA v. 0.7.17	Li & Durbin^109^	https://bio-bwa.sourceforge.net/
BWA v. 0.5.10-evan.10	Li & Durbin^109^	https://github.com/mpieva/network-aware-bwa
AuthentiCT v. 1.0.1	Peyrégne & Peter^64^	https://github.com/StephanePeyregne/AuthentiCT
mapDamage v. 2.2.3	Jónsson et al.^66^	https://github.com/ginolhac/mapDamage
R v. 4.3.3	R Core Team^110^	https://www.R-project.org/
quicksand v. 2.5	Szymanski et al.^85^	https://github.com/mpieva/quicksand
KrakenUniq v.1.0.2	Breitwieser et al.^111^	https://github.com/fbreitwieser/krakenuniq
ggplot2 v. 4.0.3	Wickham^112^	https://ggplot2.tidyverse.org/
ggrepel v. 0.9.8	Slowikowski^113^	https://github.com/slowkow/ggrepel
dplyr v. 1.2.1	Wickham et al.^114^	https://dplyr.tidyverse.org
Image Lab software v. 6.1.0	Bio-Rad Laboratories	https://www.bio-rad.com/it-it/product/image-lab-software; RRID: SCR_014210

### Experimental model and study participant details

#### Sampling of archaeological material

Sediment samples were collected in the different field sites while wearing gloves and a face mask, while animal skeletal and dental remains were taken from existing collections. For sediments, up to 100 mg of material were subsampled into fresh tubes using disposable plastic spatulas. Sampling of the skeletal and dental material was carried out using disposable dentistry drills after the removal of surface layers, collecting 50–90 mg of bone or tooth powder.

### Method details

#### Feasibility test for short DNA fragments

The immobilization capacity of various lengths and structures of DNA molecules was tested. The samples included the following: a modern, eukaryotic genomic DNA (gDNA) from *Parus major* (*P. major*); a plasmid DNA (approx. 3 kilobase pair (kb)); a 25 base-long oligonucleotide (5′-TGATACGGATAGTCCATGCTTACAG-3′); and CJ236 *E. coli* (*dut−, ung−*) gDNA, which was used as a standard in the dot-blot assay details of which are given below. We used *P. major* genomic sample as a representative of vertebrate genomic DNA (the genome sequence for *P. major* can be found at GenBank: GCA_001522545.3). DNA were isolated using the GeneJET Genomic DNA Purification kit (K0721, Thermo Fisher Scientific, USA), the NucleoSpin Plasmid Mini kit (740588.250, Macherey-Nagel, Germany) and the Quick-DNA Miniprep Plus kit (D4068, Zymo Research, USA), from *P. major*, plasmid, and *E. coli* samples respectively, all following the manufacturers’ protocols. Briefly, the samples (400, 200 and 100 ng DNA for each sample, as well as an additional 800 ng DNA for the oligonucleotide) were applied onto the prewetted, positively charged, nylon membrane (Amersham Hybond-N+; Cytiva, USA) using a vacuum-driven apparatus. The membrane was dried at 80°C for 2 h to immobilize the DNA and stained with 0.2% methylene blue followed by a wash using H_2_O. A BioRad ChemiDoc MP Imaging system was used for visualization, and the intensity values of the dots were determined using the Image Lab software (Bio-Rad, USA).

#### Dot-blot assay with ancient samples

The dot-blot experiments for uracil-DNA detection were performed using the specific sensor, 3x-FLAG-ΔUNG, following the protocol in Róna et al.^73^ with slight modifications. The 3x-FLAG-ΔUNG was produced as a recombinant protein as described in Róna et al.^73^ using the BL21(DE3) ung-151 strain^88^ to avoid active UNG contamination. Before starting the experiment, the following buffers were prepared: TE (10 mM Tris-HCl, 1 mM EDTA, pH = 8.0), ETBS-T (25 mM Tris-HCl, 2.7 mM KCl, 137 mM NaCl, 1 mM EDTA, 0.05% v/v Tween 20, pH = 7.4), and ETBS (25 mM Tris-HCl, 2.7 mM KCl, 137 mM NaCl, 1 mM EDTA, pH = 7.4).

Genomic DNA isolated from the CJ236 *E. coli* strain (*dut−, ung−*) served as uracil standard, with an initial concentration of 5 ng for the first point of the dilution series. DNA and RNA concentrations of the extracts were measured using the Qubit dsDNA HS Assay Kit (Q32851, Thermo Fisher Scientific, USA) and the Qubit RNA BR Assay Kit (Q10210, Thermo Fisher Scientific, USA), respectively, following the manufacturer’s protocol (no RNA measurement was above the detection limit). DNA extracts were then diluted starting from 0.5 μg in TE buffer (1/2 or 2/3 dilutions), corresponding to between 2.6 and 32.3 μL of extract (Table S5). We distributed 44 μL TE buffer to every empty tube, except the first with which the serial dilution starts. Then, the first tubes were prepared per DNA extract (with 0.5–1 μg), and depending on the desired dilution factor, their volume was topped up to 88 μL (1/2 dilution) or 135 μL (2/3 dilution). When making the serial dilutions, the transferred solution was mixed by pipetting for 20–30 s to attain homogeneity, and then 44 μL (1/2 dilution) or 88 μL (2/3 dilution) solution was transferred to the next tube. All serial dilutions (extract and standard) were denatured at 95°C for 5 min and put on ice for fast cooling. In parallel, the positively charged nylon membrane (Amersham Hybond-N+; Cytiva, USA) was cut in the desired size and put into TE buffer for 10 min for activation. After activating the membrane, the dot-blot apparatus (96-well Bio-Dot; Bio-Rad, USA) was assembled, and 40 μL of sample was loaded onto each well. The loaded samples were immobilized using a vacuum for a few minutes and later the membrane was transferred to a glass tray and air-dried for 15 min inside a safety cabinet. The dried membrane was left for 2 h at 80°C inside an incubator to further immobilize the samples. Five percent milk powder solution (dissolved in ETBS-T) was mixed with 50 μg/mL salmon DNA and 10 mM beta-mercaptoethanol and this blocking solution was poured onto the membrane except for a few milliliters and left for 10 min incubation. Into the remaining blocking solution, 3x-FLAG-ΔUNG sensor (in a final concentration of 12 μg/mL) was added and poured onto the membrane as well. The membrane was left for overnight incubation with agitation in a cold room (4°C). On the next day, the membrane was washed using ETBS-T buffer, enough to cover it fully, first for 3 times of 10 s and then 3 times of 10 min. A 1:10.000 dilution of the monoclonal anti-FLAG antibody (Cat. no. F3165, produced in mouse; Sigma-Aldrich, USA) prepared in 5% milk powder solution was added onto the membrane, and the membrane was incubated for 1–1.5 h at RT. The washing steps with ETBS-T buffer were repeated. A 1:10.000 dilution of the anti-*mouse*-HRP secondary antibody (Cat. no. A9044, produced in rabbit; Sigma-Aldrich, USA) prepared in 5% milk powder solution was added onto the membrane, the membrane was covered with foil and incubated for another 1–1.5 h at RT. After repeating the washing steps with ETBS-T buffer, the membrane was washed for 3 times of 10 s and then 3 times of 10 min using the ETBS buffer to remove the detergent.

To visualize the reactions on the membrane, 500 μL H_2_O was mixed with 500 μL of each of the two ECL reagents (Cat. no. WBULP, Millipore, Germany) and quickly added onto the membrane for a 1-min incubation. Then, the membrane was placed into the ChemiDoc MP Imaging System (Bio-Rad, USA) and the images of the developing blot were recorded. For each membrane, the clearest image with the least amount of oversaturation was selected and analyzed using the Image Lab software (Bio-Rad, USA). A visual workflow is presented in Figure S1B.

The uracil levels of samples were calculated using the known uracil content of the *E. coli* standard (6580 uracil/million base^73^). Intensity values of the *E. coli* dilution series were used to construct a calibration curve, using a linear regression. Intensity values from the differently-diluted samples were plotted on this calibration curve, allowing to determine the uracil-DNA content of the samples. The average uracil content of the samples was determined considering the DNA amount loaded onto the membranes, and by interpolating their intensity values onto the calibration curve.

In the pilot experiment (Test 1), we used DNA extracted from two sediment samples and two animal bones known to be positive for aDNA. After confirming the effectiveness of the method, we repeated the experiment (Test 2) using a broader set of samples, including 10 aDNA-negative extracts, 6 aDNA-positive extracts, as well as 3 extraction negative controls (aDNA preservation status was determined via hybridization capture for mammalian mitochondrial DNA (mtDNA), see below). In the final experiment (Test 3), we re-ran the available extracts to assess the reproducibility of the method.

#### Sequencing data from ancient samples

##### DNA extraction and library preparation

DNA extraction was performed following the manual version of the protocol in Rohland et al.^48^ In short, samples were incubated overnight at 37°C in 1 mL lysis buffer (0.45 M EDTA (pH 8), 0.05% (v/v) Tween 20, 0.25 mg/mL proteinase K), the supernatant were mixed with 10.4 mL binding buffer D (5 M GuHCl, 40% (v/v) isopropanol, 0.12 M sodium acetate, 0.05% (v/v) Tween 20) and loaded to silica spin columns. The columns were washed twice using 750 μL wash buffer (Buffer PE, Qiagen, Germany) and DNA was eluted in 50 μL elution buffer (0.01 M Tris-HCl (pH 8), 0.001 M EDTA (pH 8), 0.05% (v/v) Tween 20). Five or ten μL of each DNA extract were then converted to single-stranded, double-indexed DNA libraries as in Gansauge et al.^46^ Prior to PCR amplification, an aliquot of each DNA library underwent quality control assessment via two qPCR assays: one that measures the total yield of library molecules (Assay A) and the other the number of molecules generated from a spiked-in control oligonucleotide (Assay B).^46^ After PCR amplification and purification, DNA library concentrations were quantified using a NanoDrop spectrophotometer according to the manufacturer’s instructions. At least one extraction negative control and one library preparation negative control was carried along with each batch of processed samples. See details of the laboratory procedures in Table S1 and a schematic diagram in Figure S1A.

##### Targeted enrichment and sequencing

Each DNA library underwent shotgun sequencing on an Illumina NextSeq2000 platform, using paired-end sequencing with 51 + 7 cycles. In addition, an aliquot of each library underwent targeted enrichment for mammalian mtDNA using a set of probes encompassing the full mitochondrial genomes of 242 mammalian species.^89^ Two rounds of on-beads hybridization capture were performed, the first using ∼1 μg of DNA as input, and the second using ∼500 ng DNA, following the protocol of Maricic et al.^115^ as modified in Slon et al.^49^ For the samples from Perspektywiczna Cave, two such aliquots of each library underwent targeted enrichment, which were performed as separate experiments, to yield sufficient data for downstream analyses. Sequencing was performed on an Illumina MiSeq platform, using paired-end sequencing with 76 + 7 cycles. We note that the sequencing of the libraries used in this study was performed on multiple runs, with the libraries here pooled with others stemming from different projects.

### Quantification and statistical analysis

#### Raw sequencing data processing

Raw base call (BCL) files generated on the MiSeq and NextSeq 2000 platforms were converted to demultiplexed FASTQ files using Illumina BaseSpace Sequence Hub (FASTQ Generation or BCL Convert workflows, respectively) with default parameters. Then, overlapping paired-end reads were merged and the adaptor sequences were removed using AdapterRemoval version 2.3.2^106^ as implemented in nf-core/eager version 2.4.7.^107^ Resulting FASTQ files were converted to bam files using samtools version 1.19.2.^108^

#### Analysis of shotgun data

Reads were mapped using BWA (version 0.7.17) align^109^ with “ancient parameters” (bwa aln -l 1024 -n 0.04 -t 12 -o 2)^116^ to a concatenated reference including the human reference hg38 and a set of 44 microbial genomes comprising fungal and archaeal representatives together with bacterial genomes commonly associated with humans, sediments or burials (Table S2). Reads assigned to the different taxa were then examined using samtools 1.9 idxstat and separated into reads mapping to either human or microbial genomes using samtools view.^108^ Deamination estimates were first obtained using AuthentiCT version 1.0.1,^64^ while mapDamage version 2.2.3^66^ was used to compute nucleotide substitution profiles, which were plotted with R 4.3.3.^110^ Following Szymanski et al.^85^ samples were considered to contain ancient microbial DNA if the frequency of terminal cytosine to thymine substitutions to a reference genome was significantly higher than 9.5% in at least one end of fragments, based on the lower end of a binomial confidence interval.

#### Analysis of mtDNA capture data

The taxonomic identification of sequenced mtDNA fragments at the biological family level and the assessment of aDNA authenticity were performed using quicksand version 2.5^85^ with default parameters. As part of this pipeline, initial k-mer–based assignment was conducted with KrakenUniq version 1.0.2,^111^ using the mammalian mtDNA genomes in the NCBI RefSeq database release 221^117^ with k-mer size 22. Then, reads from each family were mapped using BWA version 0.5.10-evan.10^109^ to the reference genome with the highest number of assigned reads (default setting) and authenticated based on the presence of elevated C to T misincorporation patterns at the 5′- and/or the 3′ ends of fragments. A taxon was considered to be ancient if the frequency of terminal cytosine to thymine substitutions to the relevant reference genome was significantly higher than 9.5% in at least one end of fragments, as evaluated based on the lower end of a binomial confidence interval. As above for shotgun data, we used mapDamage 2.2.3^66^ to generate nucleotide substitution profiles for each taxon, which were then plotted in R 4.3.3.^110^ For the libraries from Perspektywiczna Cave for which targeted enrichment was performed twice, the bam files for each sample were merged with samtools version 1.19.2^108^ prior to the performance of the taxonomic analysis with quicksand.

As recommended in Szymanski et al.^85^ we considered two post-processing filters to reduce spurious taxonomic assignments. First, we evaluated the percentage of identified reads assigned to a given family. Applying the recommended threshold of 0.5% would have removed 19 taxa from our samples, none of which were considered to be ancient based on C to T substitution patterns. Second, we checked the evenness of sequence coverage of the reference genome (proportion of expected coverage breadth). Of the taxa deemed to be ancient, two narrowly fell below the recommended threshold of 0.5 (0.471 and 0.456 for the Hyaenidae in sample S7 and for the Ursidae in sample AB2, respectively). However, we note that both samples had low total read counts (<10,000), suggesting limited statistical power to achieve broader genome coverage. Given their damage profiles and plausible taxonomic assignments, these samples were nonetheless retained as ‘positive’ for downstream analyses.

#### Inferred uracil content and sequencing data

##### Logistic regression

Data were analyzed with a logistic regression in R version 4.3.3.^110^ Likelihood-ratio tests were performed comparing each model with the respective null-model not including inferred uracil-content as a predictor. Potential batch-effects between Test 2 and Test 3 were accounted for by estimating a regressed uracil-content per measure, taking advantage of the samples present in both batches. This per-sample batch-corrected uracil-content estimate was then used for the logistic regression to test whether it predicts the presence of aDNA, modeled as a binary response variable (samples were considered positive for aDNA if they were positive either only for ancient mammalian mtDNA; or for either ancient mammalian mtDNA or ancient microbial DNA; see Table 1).

##### Association with deaminated fragment yield

The yield - in terms of number of deaminated fragments (identified based on terminal C to T substitutions at either end) obtained per sample in the mtDNA capture data (Table S3) - was modeled with a negative binomial due to the high overdispersion (theta = 0.254), using the R function glm.nb and the following syntax: glm.nb(deaminated_reads ∼ U_content + offset(log(n_total_reads))) where deaminated_reads and n_total_reads indicate the number of fragments deaminated at the 3′ or 5′ termini and total reads per sample, respectively; and U_content indicates the number of estimated uracils per million bases.

A 0-inflated negative binomial was also run using the R package glmmTMB using the syntax: glmmTMB(deaminated_reads ∼ U_content + offset(log(n_total_reads)), ziformula = ∼ 1, family = nbinom2). However, we note that 0-inflation did not improve significantly the model, as the simpler negative binomial showed a lower AIC (130.2192 vs. 132.2192).

##### Correlation with fragments from ancient taxa

For each sediment sample, we compared the inferred uracil content obtained from the dot-blot assay (Table S5) with the total number of unique DNA fragments assigned to taxa authenticated as ancient in the mtDNA capture data (Table S3), normalized by the amount of input material into DNA extraction (Table S1). The association between these variables was tested using Spearman’s rank correlation in R version 4.3.3^110^ and data visualization was performed using the packages ggplot2^112^ v.4.0.3, ggrepel v.0.9.8^113^ and dplyr v.1.2.1,^114^ using the inferred uracil content from Test2 for all samples that were also ran on Test3. As a sensitivity analysis, the correlation was repeated after excluding samples in which no authenticated ancient mtDNA was detected. Samples for which the exact amount sampled was erroneously not recorded (S11-S13, Table S1) were excluded from this analysis.

## Supplemental information

Document S1. Figures S1–S4 and Tables S2 and S6

Table S1. Information on archaeological samples and procedures in the aDNA laboratory, related to Table 1

Table S3. Taxonomic assignment of sequenced DNA fragments and evaluation of ancient DNA preservation following targeted enrichment for mammalian mtDNA For each sample tested and associated controls, the output of quicksand v.2.5 (Szymanski et al.^85^) used for taxonomic identification and aDNA authentication is shown. Note that taxonomic interpretation was conducted at the biological family level (the “species” column indicates the reference genome used for evaluating nucleotide substitution patterns, not species-level identification). Samples and biological families determined to contain aDNA are highlighted in yellow. Families comprising less than 0.5% of identified sequences in the libraries (“FamPercentage” column) are shown in gray, and those where the expected breadth coverage (“ProportionExpectedBreadth”) is below 0.5 are marked with an asterisk. See STAR Methods for details on threshold criteria. Column headers follow the documentation on the quicksand GitHub repository, with the definitions provided within the spreadsheet.

Table S4. Evaluation of ancient microbial DNA preservation in shotgun data “Raw reads”: raw number of sequences in the file; “reads mapped human”: number of reads mapped to the human reference genome; “reads mapped microbial”: number of reads mapped to the concatenated microbial panel; 5'_total_C and 3'_total_C: the total number of informative C at the 5′ and 3′ ends of fragments, respectively; “5′ rate%” and “3′ rate%”: the rates of C-to-T substitutions at the 5′ and 3′ ends of fragments, respectively, with their lower and higher 95% binomial confidence intervals (− and +). Samples suspected of containing ancient microbial DNA are highlighted in yellow.

Table S5. Metadata of the dot-blot assays for the twenty archaeological samples tested and associated negative controls, related to Figure 2 Data in columns (L)–(Q) are shown as the mean count of uracil residues per million bases ± SD. SD, standard deviation.
