# Supplementary material for: Rapid screening of ancient DNA extracts through uracil detection
Source: iScience. 2026 Oct 15;29(11):117820. doi: 10.1016/j.isci.2026.117820 (PMC13691247; doi:10.1016/j.isci.2026.117820)
Supplement: Supplementary file 1 [file mmc1.pdf]

iScience, Volume *29*

Supplemental information

Rapid screening of ancient DNA extracts through uracil detection

Nihan Dilşad Dağtaş, Viktória Perey-Simon, Fabrizio Mafessoni, Dheeraj Chaudhary, Azhar Jabareen, Aviva Lindell, Valentina Vanghi, Krzysztof Cyrek, Andrew W. Kandel, Maciej T. Krajcarz, Magdalena Krajcarz, György Lengyel, Manuel Ángel Rojo-Guerra, Shmuel Safra, Policarpo Sánchez-Yustos, Ron Shimelmitz, Magdalena Sudoł-Procyk, Jarosław Wilczyński, Eszter Holub, Angéla Békési, Beáta G. Vértessy, and Viviane Slon

<u>**Supplementary Information**</u>

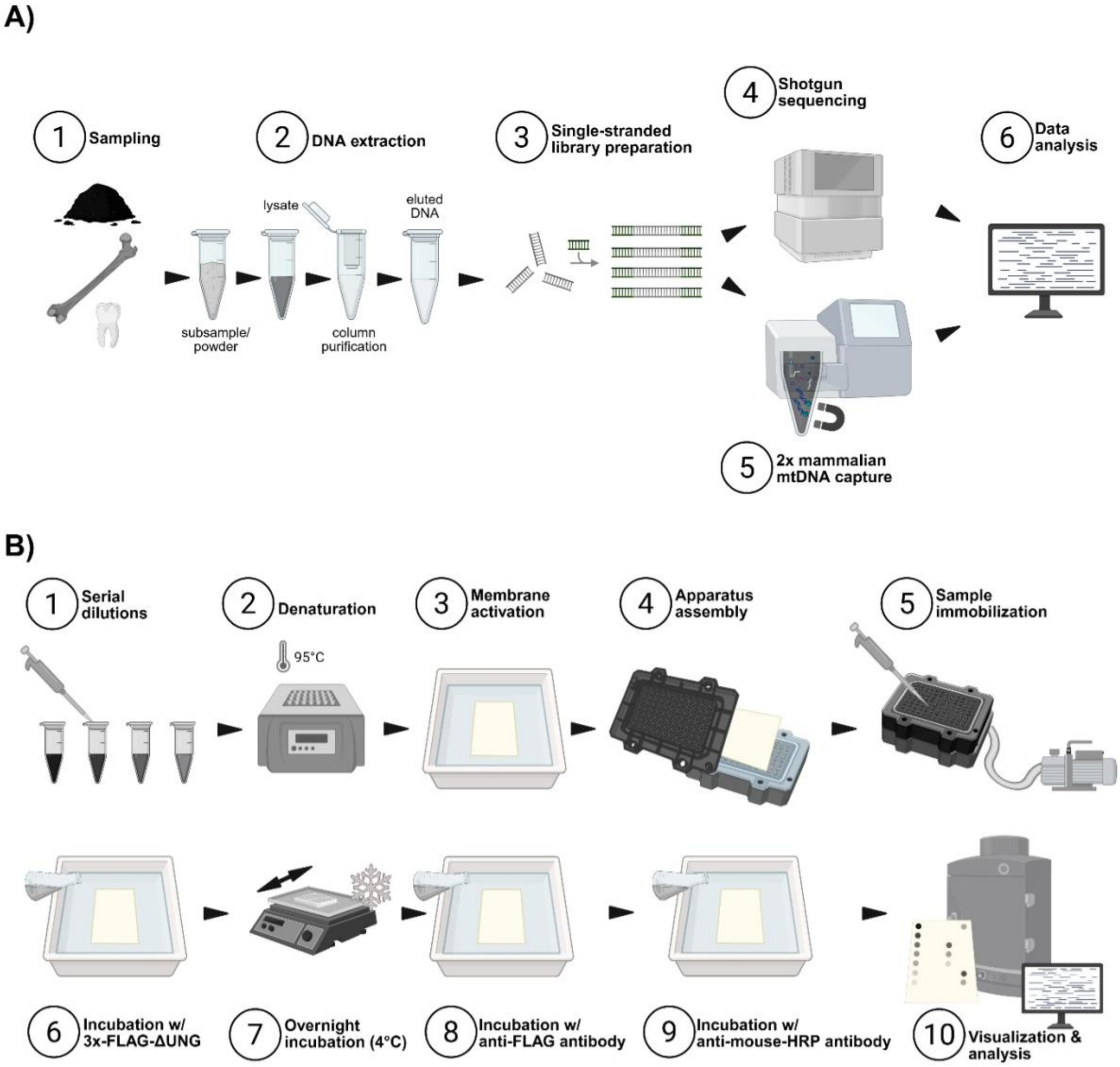

Supplementary Figure S1. Schematic experimental workflows. A) Generating genetic data from archaeological material. Steps 1-3 were carried out in a dedicated clean room. B) Performing a dot-blot assay using the generated extracts. Created in BioRender (Slon, V. (2026) https://BioRender.com/vpiu566).

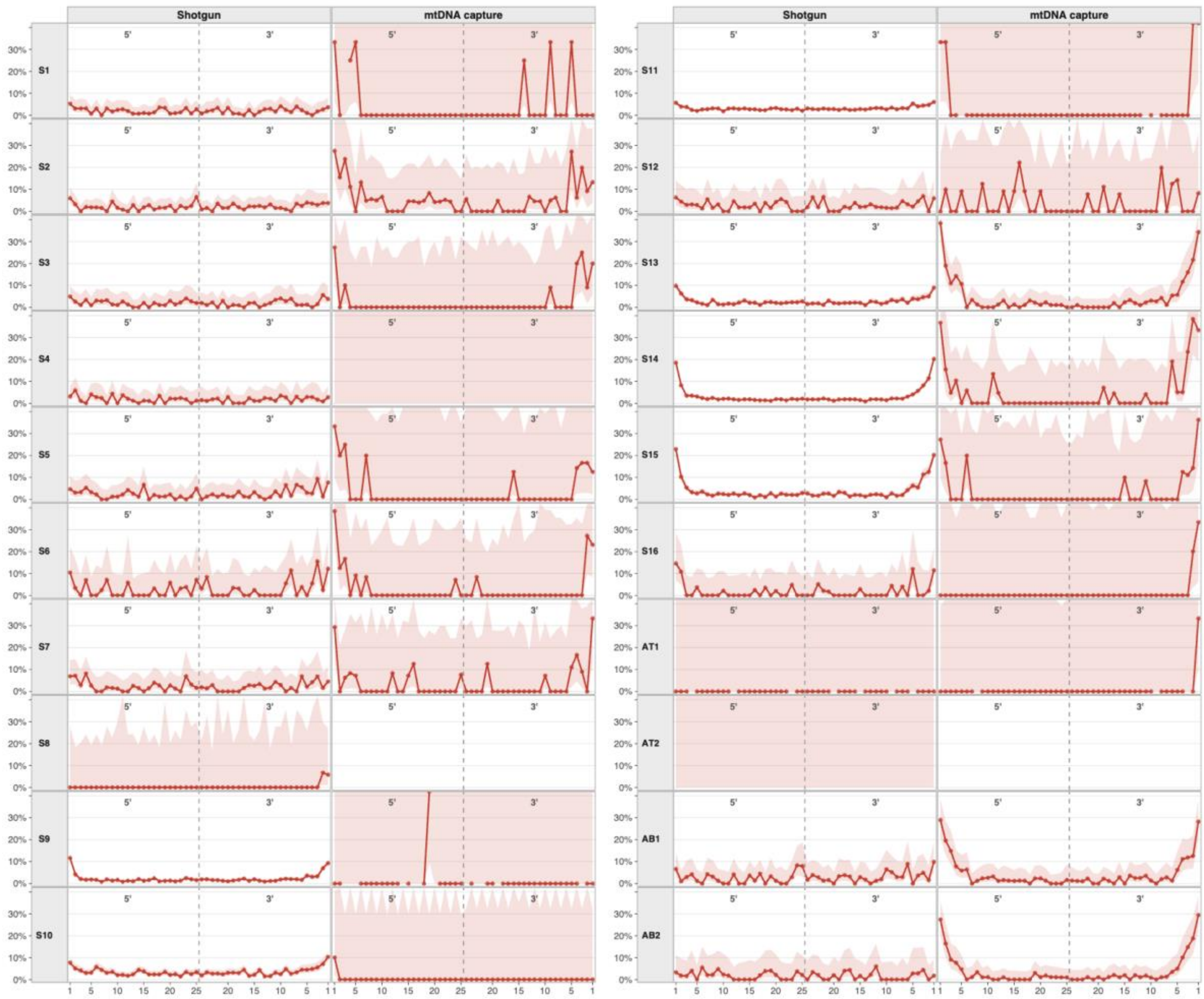

Supplementary Figure S2. Frequencies of cytosine to thymine (C to T) substitutions compared to a reference genome at the 5'- and 3'-ends of fragments. 95% binomial confidence intervals are shown in pale red. For each sample, the frequency of C to T substitutions is shown for the shotgun data compared to a concatenated reference made of 44 microbial genomes (left), and after targeted enrichment for mammalian mtDNA compared to the genome with the highest number of mapped reads (for the positive samples – out of the taxa determined to contain aDNA; for the negative samples – out of all taxa) (right).

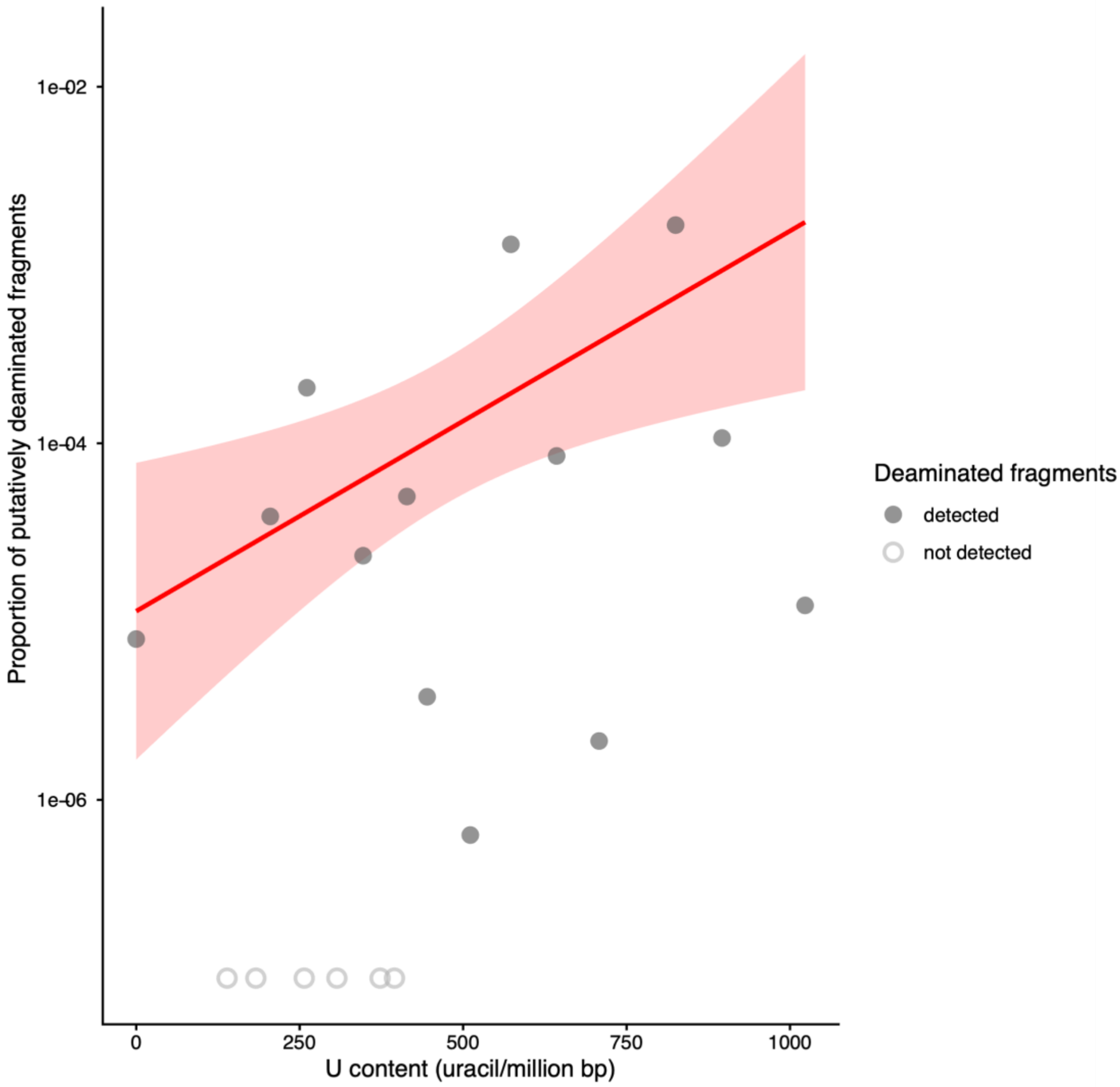

Supplementary Figure S3. Uracil content predicts the yield of deaminated fragments across extracts. Each point represents one extract, plotted as the proportion of deaminated fragments (terminal C-to-T substitution on either end of the fragment) against uracil content (uracil/million bp). Open circles indicate extracts with no deaminated fragments detected. The red line shows the fitted relationship from a negative binomial GLM with total read count as an offset to account for sequencing depth, with the shaded area representing the 95% confidence interval on the mean prediction. Proportions are displayed on a log scale.

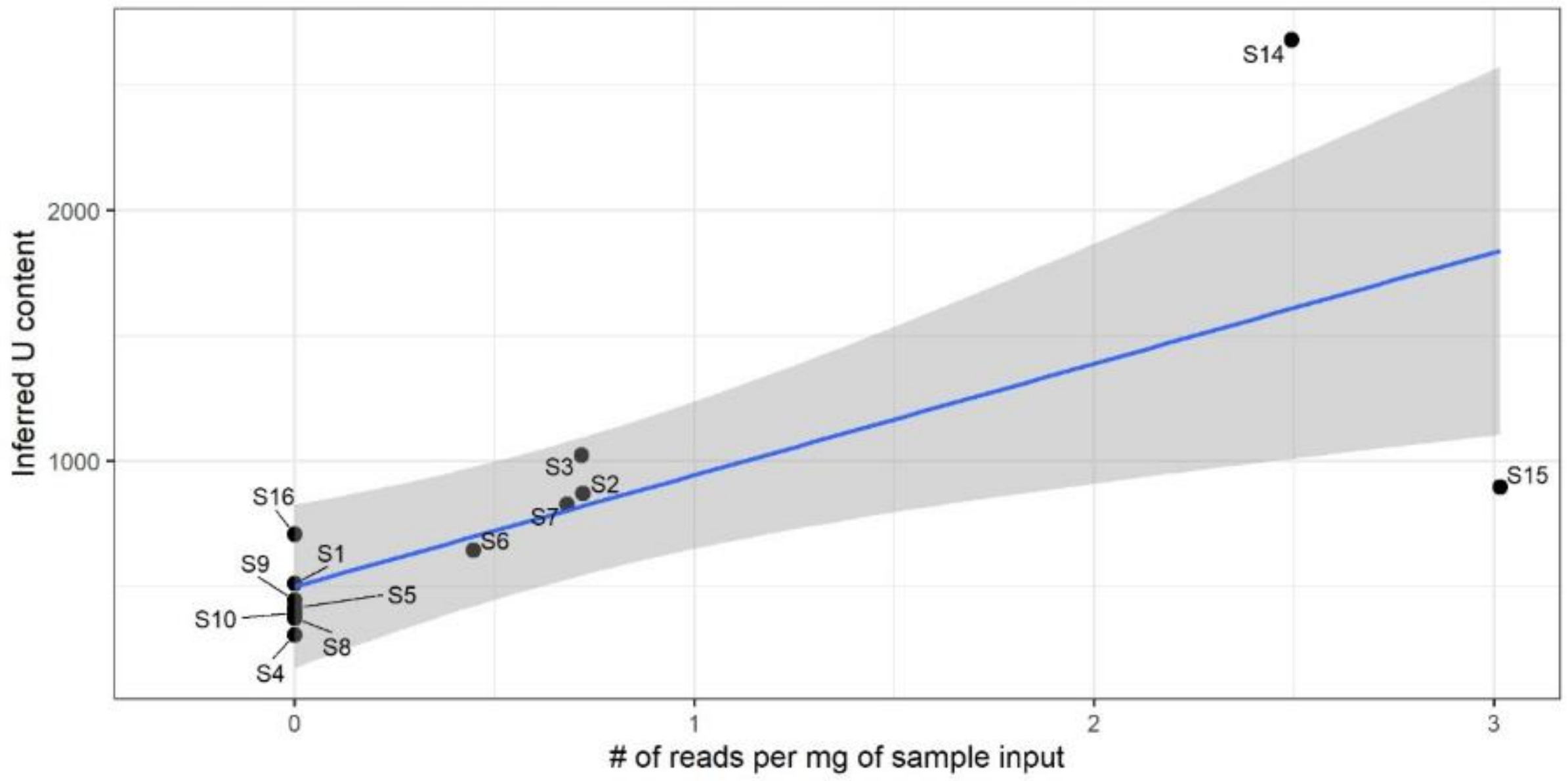

Supplementary Figure S4. Correlation between inferred uracil content and counts of DNA fragments in ancient taxa for archaeological sediment samples. The relationship between inferred uracil content determined by the dot-blot assay and the number of unique DNA fragments assigned to taxa authenticated as ancient in the mtDNA capture data, normalized by the amount of sediment input into DNA extraction. Samples without authenticated aDNA are plotted at zero. The solid line represents the fitted linear regression, and the shaded area indicates its 95% confidence interval.

Supplementary Table S2. List of microbial sequences used for the analysis of shotgun data.

**Genome**	**Taxon**
GCF_000582515.1	*Yersinia_similis*
GCF_000834215.1	*Yersinia_frederiksenii*
GCF_000834515.1	*Yersinia_intermedia*
GCF_000173735.1	*Yersinia_aldovae*
GCF_002794215.1	*Yersinia kristensenii*
GCF_000009345.1	*Yersinia enterocolitica*
GCF_000002655.1	*Aspergillus fumigatus*
GCF_000253175.1	*Yersinia enterocolitica subsp. palearctica Y11*
GCF_003253775.1	*Mycobacterium leprae*
GCF_000007565.2	*Pseudomonas putida*
GCF_000046845.1	*Acinetobacter baylyi*
GCF_003314795.2	*Sphingomonas paucimobilis*
GCF_000072485.1	*Stenotrophomonas maltophilia*
GCF_000146165.2	*Shewanella oneidensis*
GCF_000009285.1	*Cupriavidus necator*
GCF_000011365.1	*Bradyrhizobium diazoefficiens*
GCF_000203835.1	*Streptomyces coelicolor*
GCF_000008345.1	*Cutibacterium acnes*
GCF_009730415.1	*Micrococcus luteus*
GCF_000009045.1	*Bacillus subtilis str. 168*
GCF_000016785.1	*Thermotoga petrophila*
GCF_000195955.2	*Mycobacterium tuberculosis* H37Rv
GCF_000022085.1	*Methilobacterium nodulans*
GCF_000011385.1	*Gloeobacter violaceus*
GCF_000020965.1	*Dictyoglomus thermophilum*
NC_003143.1	*Yersinia pestis chr*
NC_003134.1	*Yersinia pestis pMT1*
NC_003132.1	*Yersinia pestis pPCP1*
NC_003131.1	*Yersinia pestis pCD1 shared*
NC_006155.1	*Yersinia pseudotuberculosis chr*
NC_001422.1	*PhiX174*
NC_004840.1	*Broad host plasmid PB10*
NC_004631.1	Salmonella enterica subsp. enterica serovar
NC_003198.1	*Salmonella enterica2*
NC_000962.3	*Mycobacterium tuberculosis_H37Rv*
NC_000964.3	*Bacillus subtilis*
NC_003366.1	*Clostridium perfringens*
NC_011832.1	*Methanosphaerula palustris*
NC_003888.3	*Streptomyces coelicolor* A3(2)
NC_006085.1	*Cutibacterium acnes*
NC_000913.3	*Escherichia coli K12*
NC_003450.1	*Corynebacterium glutamicum*
NC_008699.1	*Nocardioides sp.*
NC_004347.1	*Shewanella oneidensis MR-1*

Supplementary Table S6. Estimates of hands-on time, costs per sample and processing capacities for the different laboratory procedures needed to generate ancient DNA data, and for the dot-blot assay.

Step	Material used	Approximate consumable cost (*)	Number of reactions in parallel (#)	Hands-on time (@)
**Sampling**	Archaeological specimen	$5-10 per sample<br>for skeletal/dental material;<br><1$ for sediment sub-sampling	1 sample at a time	Minutes-hours<br>per sample
**DNA extraction**	Sample powder/sediment	$20-35/sample	Manual: ~12-24<br>Automated: 96	1–2 days
**DNA library preparation (including indexing PCR and quantification)**	DNA extract aliquot	$40-100/sample	Manual: ~12-24<br>Automated: 96	2-4 days
**Targeted enrichment**	DNA library aliquot	$50-250/sample	Manual: 96<br>Automated: 96-384	3-10 days
**Low-level sequencing**	DNA library pool	$5-50/sample	10s-1000s	1-2 days
**Uracil-DNA dot-blot assay**	DNA extract aliquot	$3-6/sample (~$60/membrane)	10–20<br>samples per membrane	1–2 days

* Costs are approximate and may vary substantially among laboratories, countries, and suppliers. Estimates include consumable reagents and materials only; excluding additional costs such as personnel costs, laboratory overhead, equipment purchase and maintenance, etc.

# The number of reactions includes laboratory positive and negative controls added routinely to each procedure; thus, the number does not correspond to the number of archaeological samples that can be processed at once.

@ Reported times represent approximate time for laboratory procedures, including incubations where applicable. Note that these may differ substantially from real time elapsing from sampling to first sequencing results due to delays related to working schedules, sample batching and laboratory capacity for parallelization, backlogs, shipping and queue times, and/or turnaround times at external service providers (e.g., sequencing faciltities).
